# Cardiomyocyte Marker Expression in Mouse Embryonic Fibroblasts by Cell-Free Cardiomyocyte Extract and Epigenetic Manipulation

**Published:** 2014-03

**Authors:** Tahereh Talaei-Khozani, Fatemeh Heidari, Tahereh Esmaeilpour, Zahra Vojdani, Zohrah Mostafavi-Pour, Leili Rohani

**Affiliations:** 1 Laboratory for Stem Cell Research, Department of Anatomy, School of Medicine, Shiraz University of Medical Sciences, Shiraz, Iran;; 2 Department of Tissue Engineering, School of Advance Sciences and Technology, Shiraz University of Medical Sciences, Shiraz, Iran;; 3 Department of Biochemistry, School of Medicine, Shiraz University of Medical Sciences, Shiraz, Iran

**Keywords:** Cardiomyocytes, Cell transdifferentiation, Histone deacetylase inhibitors, Fibroblast

## Abstract

**Background: **The regenerative capacity of the mammalian heart is quite limited. Recent reports have focused on reprogramming mesenchymal stem cells into cardiomyocytes. We investigated whether fibroblasts could transdifferentiate into myocardium**.**

**Methods: **Mouse embryonic fibroblasts were treated with Trichostatin A (TSA) and 5-Aza-2-Deoxycytidine (5-aza-dC). The treated cells were permeabilized with streptolysin O and exposed to the mouse cardiomyocyte extract and cultured for 1, 10, and 21 days. Cardiomyocyte markers were detected by immunohistochemistry. Alkaline phosphatase activity and OCT4 were also detected in cells treated by chromatin-modifying agents.

**Results:** The cells exposed to a combination of 5-aza-dC and TSA and permeabilized in the presence of the cardiomyocyte extract showed morphological changes. The cells were unable to express cardiomyocyte markers after 24 h. Immunocytochemical assays showed a notable degree of myosin heavy chain and α-actinin expressions after 10 days. The expression of the natriuretic factor and troponin T occurred after 21 days in these cells. The cells exposed to chromatin-modifying agents also expressed cardiomyocyte markers; however, the proportion of reprogrammed cells was clearly smaller than that in the cultures exposed to 5-aza-dC , TSA, and extract.

**Conclusion:** It seems that the fibroblasts were able to eliminate the previous epigenetic markers and form new ones according to the factors existing in the extract. Since no beating was observed, at least up to 21 days, the cells may need an appropriate extracellular matrix for their function.

## Introduction

The transdifferentiation of various cells, including somatic and adult stem cells, is a new frontier in cardiovascular research. It is also considered as a novel approach in restoring the contractile function of damaged hearts. Transdifferentiation happens in normal development^1^ and in pathologic conditions.^2^^,^^3^ Fully differentiated adult cells can transdifferentiate into other cell types by reprogramming the nucleus and cytoplasm.^4^ The reprogramming of the cells can happen in vivo^5^ or in vitro.^6^

Adult stem cells can reprogram into cardiomyocytes by various methods. It has been shown that two cardiac transcription factors, Gata4 and Tbx5, and a cardiac-specific subunit of (Brg/Brahma-associated factors) chromatin-remodeling complexes could cause the mouse mesoderm to differentiate into beating cardiomyocytes and repress the non-cardiac mesodermal genes.^7^ The fibroblast has been differentiated directly into cardiomyocytes by a combination of three cardiac-specific transcription factors in the mouse.^8^^,^^9^ Direct cell-cell contact can also induce transdifferentiation in adult stem cells. The expression of cardiomyocyte markers has been observed via the co-culturing of mesenchymal stem cells with cardiomyocytes.^10^ Blood-derived human adult endothelial progenitor cells have also been converted into cardiomyocytes through co-culturing with rat cardiomyocytes.^11^ Transdifferentiation can also be achieved by the administration of some chemicals, as has been shown by studies that report the transdifferentiation of mesenchymal stem cells into cardiomyocytes by exposure to 5-Azacytidine.^6^^,^^12^ Although the reprogrammed cells are known to have expressed cardiomyocyte markers, they are not functional in vitro*.*^13^

Factors in the cell-free extract can also induce stem cells isolated from different species to differentiate into cardiomyocytes. Human mesenchymal stem cells isolated from the bone marrow^12^ and adipose tissue^14^^,^^15^ express cardiomyocyte markers when permeabilized by streptolysin O in the presence of the rat cardiomyocyte extract. Human adipose-derived stem cells can be reprogrammed to cardiomyocytes by lipofection-mediated transfection with the cell extract from neonatal rat cardiomyocytes.^16^ Most of these studies were performed on mesenchymal stem cells.

Profound changes in gene expression are involved in cell differentiation. Epigenetic modification changes the cell fate and provides a molecular basis for cell plasticity.^17^ Chromatin-modifying agents, Trichostatin A (TSA) and 5-Aza-2-Deoxycytidine (5-aza-dC) have been shown to improve reprogramming efficiency.^18^^-^^20^ 5-Azacytidine is an analogue of a nucleoside present in DNA and RNA and can replace cytidine in DNA. It can act as an inhibitor of DNA methyl transferase. Trichostatin A is an organic component with anti-fungal properties and can inhibit the histone deacetylase enzyme family.^21^ 5-Azacytidine is known to cause mesenchymal stem cells to express cardiomyocyte markers.^6^ DNA methylation inhibitors promote the morphological transformation of myoblasts into smooth muscle cells.^22^ The in vivo administration of Trichostatin A has been shown to preserve cardiac performance.^23^

The reprogramming of differentiated somatic cells such as fibroblasts, which is easily accessible, can be considered for therapeutic use. The objective of this study was to induce the expression of cardiomyocyte markers in fibroblasts. Chromatin-modifying agents, accompanied by the cell-free cardiomyocyte extract, were used to improve the cell reprogramming efficiency.  

## Materials and Methods

This study was performed in the Laboratory for Stem Cell Research of the Anatomy Department in Shiraz University of Medical Sciences between 2010 and 2011. 

*Cell Culture*

Mouse embryonic fibroblasts (MEF) were isolated from mouse embryos on day 13 of gestation. The embryos were removed from the uterus and their conceptus was separated. Next, the heads and viscera were removed and the rest of the embryos were minced into small pieces. The minced embryos were pushed to pass through a no.18 gage needle. The cells were thereafter cultured in DMEM (Gibco) containing 15% fetal calf serum (FCS), 0.1 mM β mercaptoethanol, 1% L-glutamine, and 1% Penicillin/Streptomycin and were subcultured up to the third passage. 

*Experimental Design*

The mouse embryonic fibroblasts were aliquoted into four parts. The first culture was supplemented both extract and chromatin-modifying agents, the second one was treated with an extract without chromatin-modifying agents, and the third culture was treated with chromatin-modifying agents. The last part of the cells (control) was treated with vehicle. Chromatin-modifying agents were added to 50% confluent cells. The cells were treated with 2 µM of 5-aza-dC (Sigma) and 0.5 µM of TSA (Sigma) for 24 h. The same concentrations of TSA were added for a further 72 h.^24^ Cell viability was assessed by 0.4% trypan blue diluted with distilled water.^25^ On the 4^th^ day, the cells were harvested for permeabilization.

*Cell-Free Extract Preparation*

Cardiomyocytes were isolated from 30 mice, aged 4 to 5 weeks. The animals were killed in accordance with the Guidelines of the Ethics Committee of Shiraz University of Medical Sciences. The mice were anesthetized and their hearts were exposed. The beating hearts were perfused with cold Hanks’ balanced salt solution (HBSS) containing 3% FBS to remove the circulating blood. They were then perfused with cold HBSS containing 3% FBS and 0.1% collagenase and also HBSS containing 3% FBS and 0.1% EDTA, respectively. The perfusion took 15 min. Then, the hearts were removed and washed in cold Ca and Mg-free PBS (PBS^-^) containing 1% Penicillin/Streptomycin. The ventricles were separated from the atria and minced.^26^ The minced tissues were washed three times with PBS^-^, exposed to trypsin/EDTA at 37°C for 20 min and then centrifuged. The supernatant, which mainly contained RBC, was discarded. The pellet was snap frozen in liquid nitrogen and stored at −80°C for not longer than one month.^15^

To prepare the cardiomyocyte extract, the cells were thawed on ice and washed twice in cold PBS. An equal volume of cold lysis buffer [containing 50 mM NaCl, 5 mM MgCl_2_, 20 mM HEPES, 1 mM dithiothreitol, and protease inhibitor (Sigma)] was added to the cardiomyocytes. The mixture was incubated at 4°C for 45 min and was then sonicated (Heilscher) until all the cells and nuclei were disrupted as jugged by inverted microscopy observation. The lysate was centrifuged at 15000 g at 4°C for 15 min. The supernatant was aliquoted in 100-µL portion, snap-frozen in liquid nitrogen, and then stored at −80°C until use.^15^

The protein concentration was determined using *bicinchoninic* acid/copper sulfate assay (BSA Protein Assay Kit, Pierce) according to the manufacturer’s instructions. The concentration of protein was 8.670 mg/mL and its pH was 7.5.

*Cytotoxicity* *Assay*

The cells were exposed to the serial dilution of the extract. To do this, 50000 viable cells were incubated with 20 µL of the extract for one h at 37°C. The number of viable and dead cells was counted by trypan blue staining and hemocytometer slides. The treated cells were cultured for 24 h and then stained with neutral red. The cells were fixed with calcium formol for one min and washed with PBS. One milliliter of 0.05% neutral red (wt/vol) in PBS was added to each well and left at 37°C for 2 h. The viable cells were red after staining.

*Permeabiliztion* *of the Cells*

The harvested fibroblasts were washed three times with cold PBS^-^. The cells were resuspended in cold HBSS and aliquoted in 20000 cells per 16.4 µL. The cells were incubated at 37°C for 2 min and subsequently, 4.6µL of streptolysin O (Sigma) at a final concentration of 230 ng/mL was added and incubated at 37°C for 50 min. Twenty µL of the extract containing ATP-regenerating system [ATP, GTP, creatine phosphate, and creatine kinase (Sigma)] and 25 mM of dNTP (Sigma) were added to the cells and they were incubated at 37°C for one h. Warmed culture media (37°C) containing 2 mM CaCl_2_ was added to the cells and then transferred to 24-well tissue culture plates until they attached within 2-4 h. The culture medium was replaced by DMEM containing 15% FCS, 1% Penicillin/Streptomycin, and 1% L glutamine and left in the incubator for 1, 10, and 21 days.^27^

To assay the effects of TSA and 5-aza-dC on the expression of the cardiomyocyte markers, some untreated cells were exposed to the cardiomyocyte extract as well. For control, the TSA and 5-aza-dC-treated cells and also the untreated cells were exposed to the same volume of HBSS instead of the extract. 

*Permeabilization* *Assay*

To ensure that the cells were permeabilized effectively, the permeabilization assay was done. The assay was based on the uptake of the FITC-conjugated 70000 M_r_ Dextran (Sigma) by permeabilized cells. The uptake was detected with florescent microscopy.^28^

*Immunofluorescence* **

Cardiomyocyte markers were detected by anti-α actinin (15 µg/mL), anti-cardiac troponin T (2 µg/mL), anti-atrial natriuretic peptide (1:100 dilution), and anti-myosin-heavy-chain (1:100 dilution) antibodies (all from R&D). The secondary FITC-conjugated anti-mouse antibody (Sigma) at 1:100 dilution for anti α actinin, myosin heavy chain, and cardiac troponin T and FITC-conjugated donkey anti-rabbit antibody (Santa Cruz) for atrial natriuretic peptide with the same dilution were used. The samples were washed with PBS and fixed in 4% paraformaldehyde for 20 min. The cells were washed and incubated in PBS^-^ containing 10% goat serum, 1% BSA, and 1% triton X100 for 45 min. The primary and secondary FITC-conjugated antibodies were used for one h (each). The cells were counterstained with DAPI, mounted, and observed by fluorescence microscopy (Zeiss E600).

*Pluripotency* *Markers Detection*

The 5-aza-dC and TSA-treated cells were cultured in the embryonic stem cell culture medium in the presence or absence of LIF for 3 and 10 days. The cells were stained with anti-Oct4 antibody (R&D) at a concentration of 10 µg/mL. The secondary antibody was FITC-conjugated anti-rat antibody at a concentration of 1:200. The alkaline phosphatase activity was also assessed using the alkaline phosphatase kit (Sigma). The staining procedure was done according to the manufacturer’s instruction.

## Results

*Cytotoxicity* *Assay*

The cardiomyocyte extract was not toxic; consequently, 92.3% of the unpermeabilized cells exposed to the extract for one h were viable. The extract-treated cells as well as the control cells that were treated with only HBSS were able to grow after extract exposure. Neutral red staining revealed that the cells were viable after culturing for 24 h.

*Permeabilization* *Assay*

FITC-dextran uptake was observed in the cells that were exposed to this marker in the presence of 230 ng/mL of streptolysin O. The cells treated with FITC-dextran without permeabilization with streptolysin O were also able to uptake the marker; however, the fluorescence intensity and the number of the cells that showed fluorescence were less than those in the streptolysin O-treated cells. This may be related to endocytosis, which took place during the incubation, as has been reported by other researchers.^13^ The cells were allowed to culture for 24 h. The cells were able to expand and survive, as was indicated by the neutral red assay.

*Cell Morphology*

The administration of both 5-aza-dC and TSA reduced cell growth, as was indicated by the number of the passages. Also, 5-aza-dC, when treated alone, had no influence on cell growth. Extensive cell death was observed with TSA exposure. Although the viable cells were able to proliferate, the confluency was not more than 50%. These chromatin-modifying agents also changed cell morphology. The treated cells were larger than those cultured in the absence of chromatin-modifying agents. The number of processes was reduced, and the cells were polyhedral in shape. More conspicuous morphological modification was observed in the other cell types such as human granulosa cells and mouse fibroblast cell line (NIH3T3): they became fusiform as a result of treatment with the chromatin-modifying agents. (Data are not shown.) 

**Figure 1:**
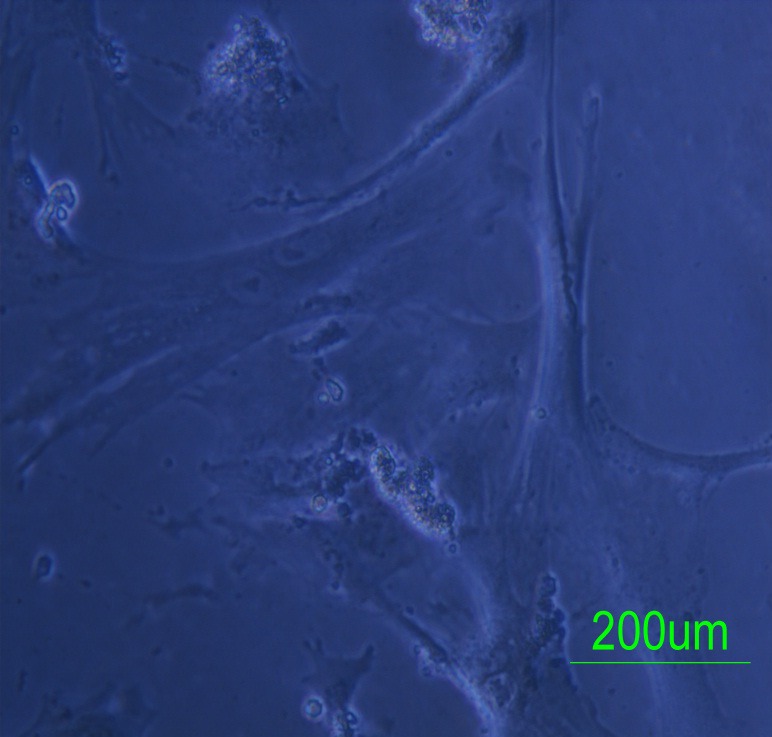
Cells treated with the extract and chromatin-modifying agents were multinucleated.

After extract treatment, more cells showed morphological changes. The cells became elongated and lost their processes. Some multinucleated cells with two or three nuclei were also observed (figure 1). There was no beating cell in the culture with this condition. The cell proliferation rate reduced significantly; however, the cells were viable for at least 30 days. While the cells in the control groups needed to passage every 3 days, the extract treated cells were not confluent even after 30 days from the beginning of the exposure to the cardiomyocyte extract.

*Cardiomyocyte* *Markers Expression*

Immunofluorescence detected the existence of cardiomyocyte markers in the fibroblasts that were exposed to chromatin-modifying agents and permeabilized in the presence of the cardiac extract. After 24 h, very small percentages of the cells treated with the extract and chromatin-modifying agents reacted with α-actinin and myosin heavy chain (2.09% and 1.97%, respectively), while only 0.4% and 1.59% of the cells expressed cardiac troponin T and atrial natriuretic peptide (table 1).

**Table 1:** Percentages of the cells that showed positive reaction to various cardiomyocyte markers

**Markers**		**Cells treated with both extract and chromatin-modifying agents (%)**	**Cells treated with extract without chromatin-modifying agents (%)**	**Cells treated with chromatin-modifying agents (%)**	**Cells without any treatment (%)**
After 24 hours	Alpha actinin	2.09	1.92	1.2	0.8
	Myosin heavy chain	1.97	1.84	0.93	0.72
	Atrial natriuretic peptide	1.59	0.62	0.71	0.6
	Cardiac troponin	0.4	0.41	0.54	0.43
After 10 days	Alpha actinin	76	12	20.3	1.7
	Myosin heavy chain	64.9	9.7	17.6	3.5
	Atrial natriuretic peptide	1.3	1.2	1.4	0.9
	Cardiac troponin	7.3	2.8	2.2	1.3
After 21 days	Alpha actinin	75	23	35	3
	Myosin heavy chain	67.9	18	20	5
	Atrial natriuretic peptide	50	9.3	2	1
	Cardiac troponin T	43.7	12.2	3.1	1

**Figure 2:**
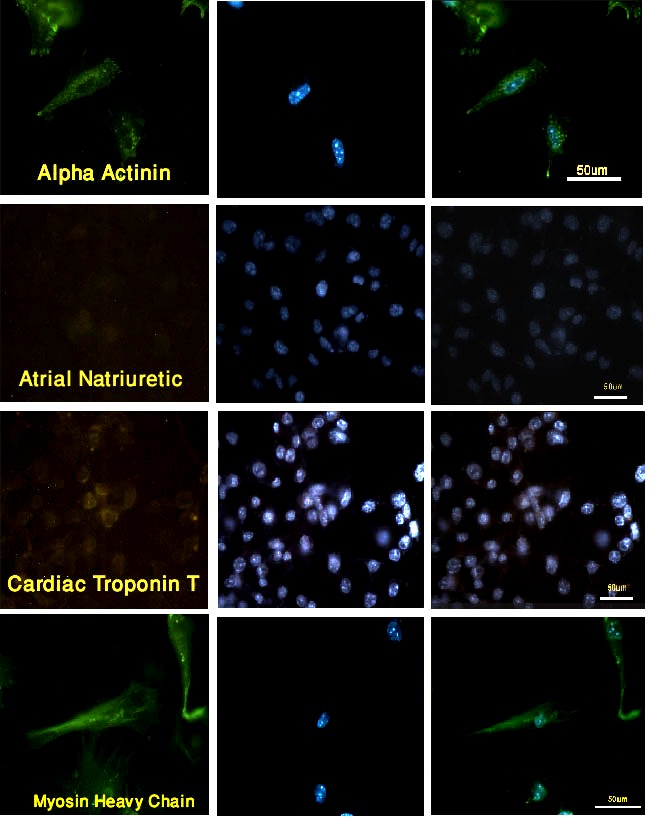
Extract and chromatin-modifying-agents-treated cells expressed myosin heavy chain and α-actinin but not atrial natriuretic peptide and cardiac troponin after 10 days. FITC (left), DAPI (middle), and Merged (right)

After 10 days, the percentages of the α-actinin and myosin-heavy-chain-positive cells treated with both extract and chromatin-modifying agents were higher than before so that 76% and 64.9% of the fibroblasts reacted with antibodies against these markers, respectively. However, just 7.3% and 1.3 % of the cells expressed cardiac troponin T and atrial natriuretic peptide (figure 2). In the cultures exposed to 5-aza-dC and TSA but not to the cardiac extract, the fibroblasts also expressed myosin heavy chain and α-actinin, although the percentage of such cells was less than that of the cells treated with the extract (17.6% and 20.3%, respectively). In the cultures exposed to chromatin-modifying agents, 1.4% and 2.2% of the cells expressed atrial natriuretic peptide and cardiac troponin, respectively. Meanwhile, 1.4 % and 2.2% of the cells permeabilized in the presence of the cardiomyocyte extract expressed atrial natriuretic peptide and cardiac troponin, respectively (table 1). The antibodies did not react with the untreated cells.

**Figure 3:**
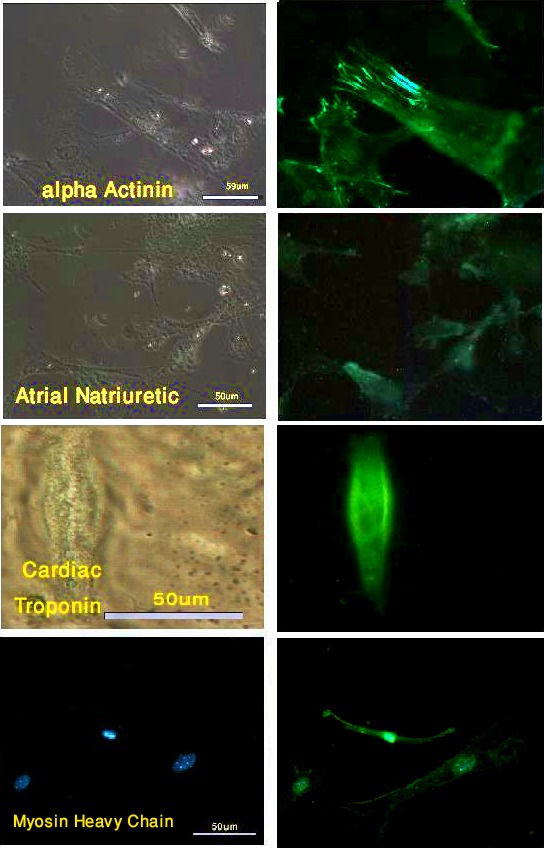
Extract and chromatin-modifying-agents-treated cells expressed all cardiomyocyte markers after 21 days. FITC (left), Phase contrast (right)

Twenty-one days after the extract treatment, a higher percentage of the cells expressed cardiac troponin and atrial natriuretic peptide (50% and 43.7%, respectively), while no change was observed in the percentage of α-actinin and myosin-heavy-chain-positive cells (67.9% and 75%, respectively) (figure 3). In the cultures only permeabilized in the presence of the cardiomyocyte extract, 23%, 18%, 9.3%, and 12.2% of the cells expressed α-actinin, myosin heavy chain, atrial natriuretic peptide, and cardiac troponin, respectively. Although the fibroblasts that were exposed to the chromatin-modifying agents were able to express myosin heavy chain and α-actinin after 21 days (20% and 35%, respectively), the expression of the other markers was negligible. The expressed markers showed a parallel arrangement in most of the reacting cells. The untreated cells expressed negligible amounts of cardiomyocyte markers at 21 days after the beginning of the experiment as well as at the other period times (table 1).

*Expression of Pluripotent Markers*

**Figure 4:**
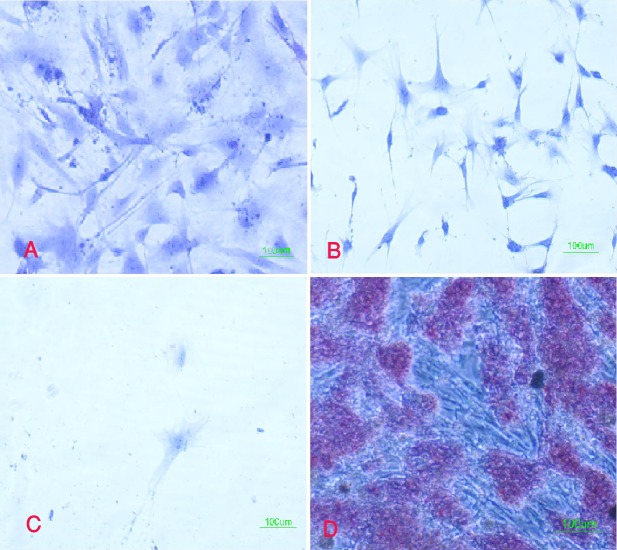
Fibroblasts exposed to chromatin-modifying agents showed no alkaline phosphatase reaction. A; untreated &lunderline&fibroblasts; B, fibroblasts that were treated with Trichostatin A and 5-Aza-2-Deoxycytidine and were cultured in the presence of LIF; C, fibroblasts that were treated with Trichostatin A and 5-Aza-2-Deoxycytidine and were cultured in the absence of LIF; D, mouse embryonic stem cell. Red cells show alkaline phosphatase activity.

The expression of pluripotency markers such as Oct4 and alkaline phosphatase was also studied in the chromatin-modifying-agent-treated cells in the presence or absence of LIF after 3 and 10 days. The data revealed that the cells could not express these two markers whether they were exposed to LIF or not. They also could not form embryonic stem cell-like colonies in the presence of the chromatin-modifying agents (figure 4).

## Discussion

There are some approaches that are capable of inducing cardiomyocyte differentiation from various types of stem cells^5^^-^^7^^,^^10^^,^^11^ with low efficiency.^29^ It has been shown that the extracts from the differentiated cells change the fate of the other cell types.^30^ Research has indicated that the extract can promote cell reprogramming in somatic cells such as fibroblasts,^6^^,^^12^^,^^28^ lymphocytes,^31^ and granulosa cells.^32^ The reprogramming of fibroblasts into insulin-producing cells by exposure to the insulinoma cell line extract has also been reported.^33^ The uptake of transcription regulators in the extract causes the cell fate to change.^34^ This study revealed that fibroblasts were also able to express cardiomyocyte markers by extract treatment. Earlier studies have shown that MSCs can differentiate into cardiomyocytes after exposure to an extract of adult mouse heart cells.^6^^,^^15^

We observed that some extract-treated fibroblasts were multinucleated; this is in agreement with other studies that showed MSCs could become multinucleated by extract exposure due to differentiation toward a myogenic phenotype.^15^^,^^12^ Cell enlargement was also observed in our experiments after extract treatment. An increase in cell size has also been reported previously in the cardiomyocyte differentiation process induced by 5-Azacytidin^6^^,^^12^ and cardiomyocyte extract^10^ in MSCs.

According to our data, the extract was able to induce the expression of cardiomyocyte markers. After 24 h, only 2.09% of the cells expressed α actinin. These cells may uptake the proteins from the extract; however, the half life of the proteins is limited. After 10 days, the extract-treated cells were able to express α-actinin and myosin heavy chain, but not the other markers. After 21 days, a high percentage of the extract-treated cells were able to express all the cardiomyocyte markers. The same results were obtained by Gaustad et al.^15^ (2004), who also used a rat cardiac extract to modify MSCs into cardiomyocytes; nevertheless, the percentage of the cells that expressed cardiomyocyte markers was higher than that we observed in the present study, probably because of the different cell types employed.

The treatment of fibroblasts with chromatin-modifying agents increases the percentage of the cells that express cardiomyocyte markers. The percentage of the cells that express cardiomyocyte markers is nearly similar to that of the cells exposed to the extract alone. The administration of chromatin-modifying agents can improve the efficiency of cell reprogramming.^19^^,^^20^^,^^35^^,^^36^ We also showed that TSA and 5-aza-dC were able to increase the percentage of the permeabilized cells that expressed cardiomyocyte markers. It has also been shown that 5-Azacytidine may activate the expression of myogenetic genes such as MyoD secondary to hypomethylating of DNA.^37^

It has been previously reported that the administration of a combination of TSA and 5-aza-dC can induce dedifferentiation in a fibroblastic model so that the embryonic stem cell markers can be expressed.^38^ We hypothesized that chromatin-modifying agents may induce fibroblasts to dedifferentiate and express pluripotency markers. The dedifferentiated cells can then differentiate into cardiomyocytes spontaneously.^39^ Therefore, we checked the expression of pluripotency markers in the fibroblasts in both the presence and absence of LIF. The results revealed that the cells could not express any pluripotency markers. Accordingly, the expression of the cardiomyocyte markers via the exposure of the cells to TSA and 5-aza-dC should be related to other factors such as the expression of the myogenic genes following epigenetic modification. Although chromatin-modifying-agents-treated cells cannot express all cardiomyocyte markers, the treatment with the extract seems to be necessary for transdifferentiation.

## Conclusion

The administration of the extract was able to induce the expression of cardiomyocyte markers. The exposure of the cells to TSA and 5-aza-dC was also able to induce the expression of cardiomyocyte markers. The treatment of the cells with a combination of the extract and chromatin-modifying agents increased the percentage of the cells expressing these markers. It seems that the chromatin-modifying agents were able to eliminate the previous epigenetic markers and form new ones according to the factors existing in the extract. No beating was observed, at least up to 21 days. We would suggest that an appropriate extracellular matrix be utilized to functionalize the cells.
